# Sea Cucumber Hydrolysates Alleviate Cognitive Deficits in D-Galactose-Induced C57BL/6J Aging Mice Associated with Modulation of Gut Microbiota

**DOI:** 10.3390/foods14111938

**Published:** 2025-05-29

**Authors:** Han Gong, Hang Zhao, Xueying Mao

**Affiliations:** Key Laboratory of Functional Dairy, Ministry of Education, College of Food Science and Nutritional Engineering, China Agricultural University, Beijing 100083, China; gonghan@cau.edu.cn (H.G.); zhaohang980813@163.com (H.Z.)

**Keywords:** sea cucumber hydrolysate, aging, cognitive deficit, gut microbiota, neuroinflammation

## Abstract

As the global elderly population is rising, concerns about cognitive decline and memory loss are becoming urgent. This study evaluated the potential of sea cucumber hydrolysates (SCH) from *Stichopus japonicus* in alleviating cognitive deficits using a D-galactose-induced murine aging model. The effects of SCH on behavior, hippocampal morphology, gut microbiota, hippocampal cholinergic system, brain-derived neurotrophic factor (BDNF) signaling, and neuroinflammatory pathways were investigated. Results showed that SCH ameliorated learning and memory deficits and reduced neuronal damage in aging mice. SCH also modulated gut microbiota, along with increased fecal short-chain fatty acids levels. Functional prediction revealed that alterations in gut microbiota were related to signal transduction. Further, SCH enhanced hippocampal cholinergic function through elevating acetylcholine (ACh) levels and inhibiting acetylcholinesterase (AChE) activity and activated BDNF signaling, consistent with predictions of gut microbiota function. Restoration of cholinergic homeostasis and transmission of the BDNF pathway might contribute to the inhibition of hippocampal neuroinflammation via suppressing microglial activation and the nuclear factor kappa-B (NF-κB) pathway. In summary, SCH attenuated cognitive deficits through suppressing neuroinflammation, which might be correlated with the signal transduction caused by regulating gut microbiota. Further validation will be conducted through microbiota depletion and fecal microbiota transplantation. These findings suggest that SCH is a promising functional component for counteracting aging-related cognitive deficits.

## 1. Introduction

Aging disrupts physiological homeostasis and accelerates neurological decline. Unfortunately, the trend of aging of the world population is intensifying rapidly, with the cohort aged ≥65 years projected to exceed 800 million by 2025 and double by 2050 [1]. The brain is one of the organs most affected by aging, and brain aging particularly drives cognitive impairments and dementia, while current pharmacological interventions often cause side effects such as vomiting and decreased appetite [2]. Hence, it is essential to explore natural alternatives targeting fundamental mechanisms.

The cholinergic system is central to cognitive function, with acetylcholine (ACh) regulating synaptic plasticity and neurotransmission. However, aging induces overactivity of acetylcholinesterase (AChE), which degrades ACh and exacerbates neuroinflammation through microglial activation and proinflammatory cytokine release [3,4]. In addition, brain-derived neurotrophic factor (BDNF) and its downstream molecule tropomyosin receptor kinase B (TrkB) could support neurogenesis, synaptic plasticity, and neuronal recovery [5]. Hence, modulating cholinergic homeostasis and activating BDNF signaling pathways represent critical therapeutic strategies to suppress neuroinflammation, thereby alleviating cognitive deficits. Compounding these neural mechanisms, emerging evidence implicates gut microbiota dysbiosis as a key contributor to neuroinflammation and cognitive decline [6,7,8]. Aging-related shifts in microbial composition disrupt intestinal barrier integrity, permitting systemic lipopolysaccharide (LPS) translocation that primes microglial activation [9]. Conversely, microbiota-derived beneficial metabolites counteracted inflammation by downregulating proinflammatory cytokines and suppressing the nuclear factor kappa-B (NF-κB) pathway [10]. Consequently, maintaining gut homeostasis is emerging as a potential strategy to support brain health in the elderly.

As nutrient-dense marine organisms, sea cucumbers represent a rich source of bioactive constituents including saponins, fucosylated chondroitin sulfate, and antioxidant phenolics [11]. Additionally, they contain proteins that can be enzymatically hydrolyzed to generate bioactive peptides. Gly is the main amino acid in sea cucumber protein, followed by Glu, Pro, and Ala, yet notably deficient in Cys [12]. Sea cucumber hydrolysates (SCH) exerted angiotensin-I-converting enzyme inhibitory effects [13]. SCH also exhibited anti-inflammatory and antioxidant properties via modulation of c-Jun N-terminal kinase signaling and sirtuin 3/superoxide dismutase [14,15]. Neuroprotective peptides from terrestrial sources such as dairy, walnuts, and seafoods have been extensively characterized [16]. Notably, SCH improved neuronal morphology and reduced AChE activity in cognitive impairment models [17,18]. However, the effects of SCH in modulating gut microbiota, thereby regulating related signaling pathways and suppressing neuroinflammation due to the amino acid composition in the peptides produced by specific enzymes, which ultimately improve cognitive deficits in aging mice remain undefined. We hypothesized that SCH supplementation could alleviate cognitive deficits in aging mice, which might be correlated with restoring gut microbiota homeostasis to induce signal transduction and suppress neuroinflammation.

Therefore, this study systematically evaluate SCH’s anti-aging-related cognitive benefits. The amino acid composition and molecular weight profile of SCH and its effects on behavior and brain morphology in aging mice were examined. Then, the impact of SCH on gut microbiota and fecal short-chain fatty acids (SCFAs) was also investigated. Additionally, cholinergic function (AChE activity, ACh content, α7 nicotinic ACh receptor (α7 nAChR) levels), BDNF/TrkB signaling, and neuroinflammation (microglia activation and NF-κB signaling) were determined in the hippocampus. This research expects to provide a theoretical support for the potential application of SCH in alleviating aging-related cognitive deficits, and the relevant mechanisms from the perspective of regulating the gut microbiota will be explained.

## 2. Materials and Methods

### 2.1. Preparation of SCH

The body walls of sea cucumbers (*Stichopus japonicus*) were cut into small pieces and thoroughly crushed with a beater and then dispersed in deionized water. The suspension was subsequently enzymatically hydrolyzed for 4 h at 50 °C with a combination of neutral protease, alcalase, and papain (Novozymes Biologicals Inc., Bagsværd, Denmark), and each enzyme was added at an amount of 3000 U/g. Then, to terminate the reaction, hydrolysates were boiled at 85 °C for 15 min. Subsequently, SCH aqueous solutions were passed via the ultrafiltration membrane (molecular weight cutoffs of 10 kDa) (Whatman, Co., Ltd., Maidstone, UK) and filtrates were freeze-dried and for subsequent experiment. All the raw materials were from the same batch of sea cucumbers, avoiding the interference of individual differences on the results.

### 2.2. Animals and Experimental Design

A total of 60 male C57BL/6J mice of eight weeks were obtained from Beijing Vital River Company (Beijing, China). They were maintained under controlled conditions (a 12:12 light-dark period, 22 ± 1 °C) and received water and standard feed ad libitum. The experimental were conducted following the Guidelines of Experimental Animals in the People’s Republic of China, and experiments were supported by the Animal Ethics Committee of China Agricultural University (the ethical review serial is AW41213202-4-1).

All mice were separated into six groups, with 10 mice in each group after 1-week acclimatization: (i) mice injected with normal saline and orally administrated with normal saline (both were 0.1 mL/10 g) were regarded as the normal group (NC); (ii) mice intraperitoneally injected with 150 mg/kg·body weight (BW) of D-galactose (Sigma-Aldrich Inc. (St. Louis, MO, USA)) and orally administrated with normal saline (0.1 mL/10 g) were regarded as the D-galactose (D-gal) group; (iii–v) mice intraperitoneally injected with 150 mg/kg·BW of D-gal and orally administrated with SCH at 200, 400, and 800 mg/kg·BW were regarded as the SCH-L, SCH-M, and SCH-H groups, respectively; and (vi) mice intraperitoneally injected with 150 mg/kg·BW D-gal and orally administrated with 1 mg/kg·BW donepezil (MedChemExpress Co., Ltd., Monmouth Junction, NJ, USA) were regarded as the positive control group [19]. Intraperitoneal injection was performed after daily intragastric administration. After 9 weeks of treatment, behavior tests were conducted and feces were collected. Subsequently, the mice were euthanized, then blood samples were centrifuged and the serum was stored at −80 °C. Moreover, the hippocampus was isolated from some brains, fixed in 4% paraformaldehyde (Solarbio Company, Beijing, China) and frozen at −80 °C, respectively.

### 2.3. Serum Biochemical Analysis

Serum levels of LPS, IL-1β, IL-6, IL-10, TNF-α, and superoxide dismutase (SOD) were detected using ELISA kits from Dogesce Biotechnology, China and Nanjing Jiancheng Company (Nanjing, China).

### 2.4. Behavioral Tests

#### 2.4.1. Y-Maze Test

The Y-maze test was conducted according to former described [20]. Mice were acclimated to the testing room for 1 h under 30 lx lighting before behavioral assessment. Each mouse was placed at the maze center and allowed to explore freely for 8 min, with arm entry sequences video-tracked. Alternation rate was defined as entering all three arms alternately in an alternating manner. Cognitive function was evaluated via the following formula: Spontaneous alternation (%) = [(number of alternations)/(total arm entry numbers-2)] × 100.

#### 2.4.2. Novel Object Recognition Test

This test was performed following a 3-phase protocol: (1) Habituation: 10 min free exploration in an empty apparatus; (2) Familiarization (24 h later): 10 min exposure to two identical objects placed in opposite corners; (3) Test (24 h post-familiarization): 5 min exploration with one familiar object replaced by new one. Exploration time (sniffing/touching) for novel (Tn) and familiar (Tf) objects was recorded. Recognition index was calculated as (Tn/Tf) × 100% [21].

#### 2.4.3. Morris Water Maze Test

The experiment was performed in a circular pool (1.5 m diameter) with water maintained at 21 ± 1 °C. A hidden platform was submerged 1 cm below the water surface, with visual cues on the pool walls and water rendered opaque using white dye. The protocol included: (1) A 5-day navigation training phase with four daily trials from randomized starting points, recording escape latency (90 s cutoff; guided to the platform if unsuccessful to find the platform), and (2) a spatial probe trial on day 6, during which the platform was removed and time spent in aimed quadrant and platform crossings was quantified in a 60 s free swim [21].

### 2.5. The H&E Staining

The H&E staining of brain tissues was conducted as former described [22]. Paraffin-embedded hippocampal sections were dewaxed in xylene, stained with hematoxylin followed by eosin counterstaining (SenBeiJia Biological Technology Company, Nanjing, China). After dehydration, slides were sealed with neutral gum (Yuanye Bio Company, Shanghai, China) and imaged using an Olympus optical microscope (Tokyo, Japan).

### 2.6. Fecal DNA Extraction, 16S rRNA Amplification, and MiSeq Sequencing

Fecal DNA was extracted using the E.Z.N.A.^®^ Soil DNA Kit (Omega, Norcross, GA, USA), and the bacterial 16S rRNA V3–V4 regions were amplified via PCR with standard thermal cycling conditions including denaturation, annealing, and extension steps [23]. PCR products were purified with a Gel Extraction kit (Axygen, Union City, CA, USA), and DNA concentrations were measured using QuantiFluorTM-ST (Promega Corporation, Madison, WI, USA).

### 2.7. Bioinformatics Analysis

Following quality filtering, raw sequences were grouped into operational taxonomic units (OTUs) with a 97% sequence similarity threshold applied using QIIME and USEARCH. Taxonomic classification was employed using the RDP classifier. Alpha diversity (Chao, Shannon, Simpson, Pielou_e indices) was calculated, while PICRUSt was predicted functional profiles through KEGG pathway (class 3) mapping.

### 2.8. Fecal SCFAs Analysis

Fecal SCFAs were analyzed by GC-MS (equipped with an Rtx-Wax column) following homogenization, acidification with 5% sulfuric acid (*v*/*v*), and ethyl ether extraction. Samples were centrifuged and supernatants were analyzed under standard GC-MS conditions with helium carrier gas at 1.0 mL/min [24].

### 2.9. Measurement of ACh Content and AChE Activity

Hippocampal tissue samples were homogenized in ice-cold normal saline based on tissue homogenizer. After centrifugation, supernatants were collected and analyzed for ACh content and AChE activity via assay kits of Nanjing Jiancheng (Nanjing, China).

### 2.10. Western Blot

Hippocampal proteins were extracted via the RIPA buffer with protease/phosphatase inhibitors, then centrifugation for 15 min at 4 °C. Equal protein amounts were separated on 10% SDS-PAGE gels, transferred to PVDF membranes, and blocked. Membranes were incubated overnight at 4 °C with anti-AChE, anti-α7 nAChR, anti-ionized calcium-binding adapter molecule 1 (IBA-1), p-inhibitor of kappa B kinase (p-IKK) α/β, IKKα, IKKβ, p-inhibitor kappa B alpha (p-IκBα)/IκBα and NF-κB p-p65 and NF-κB p65, anti-BDNF, anti-TrkB, and anti-β-actin (Abcam, Cambridge, UK) antibodies. After incubation with HRP-conjugated secondary antibodies (room temperature), bands were visualized using chemiluminescence and analyzed with Image J software (ver. 1.52a, Wayne Rasband, National Institutes of Health, Bethesda, MD, USA).

### 2.11. Molecular Weight (Mw.) Distribution Measurement

Molecular weight distribution was analyzed by HPLC (equipped with a TSK gel 2000 SWXL column (Tosoh Corporation, Tokyo, Japan) and UV detector at 220 nm). Samples were dissolved in 45% acetonitrile/0.1% trifluoroacetic acid, and molecular weights were determined using a calibration curve with protein/peptide standards (189–12,384 Da) [25].

### 2.12. Determination of Amino Acid Composition of SCH

SCH was mixed with 6 mol/L HCl, and hydrolyzed at 110 °C. After raising the pH to 7.0, the mixture was detected via amino acid analyzer (L-8900, Tokyo, Japan) to identify the composition of SCH [26].

### 2.13. Statistical Analysis

The data were presented as mean ± SEM. The statistical analysis was conducted via one-way ANOVA, and Tukey’s multiple comparison test was performed on SPSS 20.0 software. A criterion of *p* < 0.05 was applied for hypothesis testing. All data collection and analyses were performed without knowledge of treatment conditions to minimize potential bias.

## 3. Results

### 3.1. Composition Analysis of SCH

**Figure 1:**
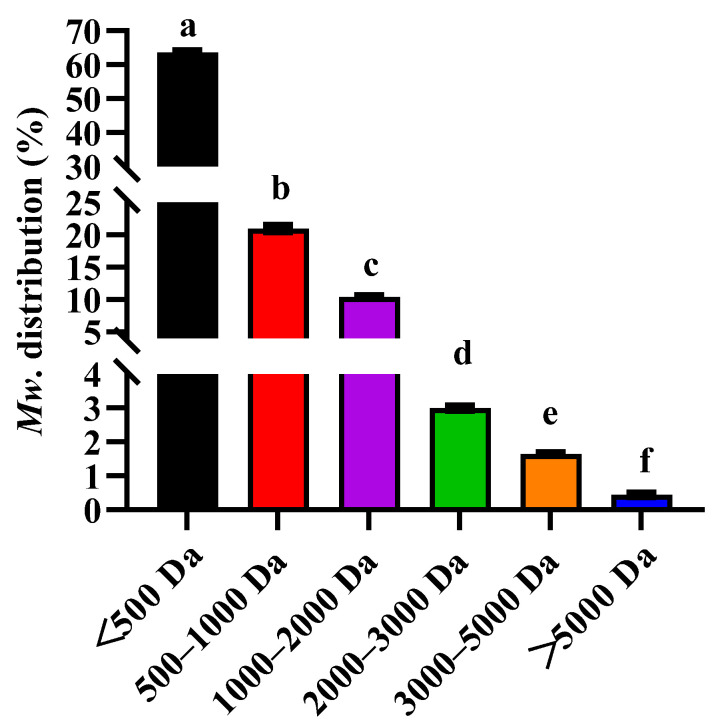
Molecular weight distribution of SCH. Letters correspond to significant difference (*p* < 0.05) (*n* = 3). SCH: sea cucumber hydrolysates.

Chromatographic analysis revealed that the molecular weight distributions of SCH obtained by enzymatic hydrolysis were as follows: 63.58% ± 0.45% of <500 Da, 20.97% ± 0.45% of 500–1000 Da, 10.40% ± 0.15% of 1000–2000 Da, 2.99% ± 0.66% of 2000–3000 Da, 1.64% ± 0.04% of 3000–5000 Da, and 0.44% ± 0.05% of >5000 Da. This result demonstrated that SCH mainly consisted of small molecular weight peptide (Figure 1). SCH contained a rich variety of amino acids, among which Gly, Glu, and Pro have higher contents (Table 1).

### 3.2. Effects of SCH on Cognitive Behaviors in Aging Mice

**Figure 2:**
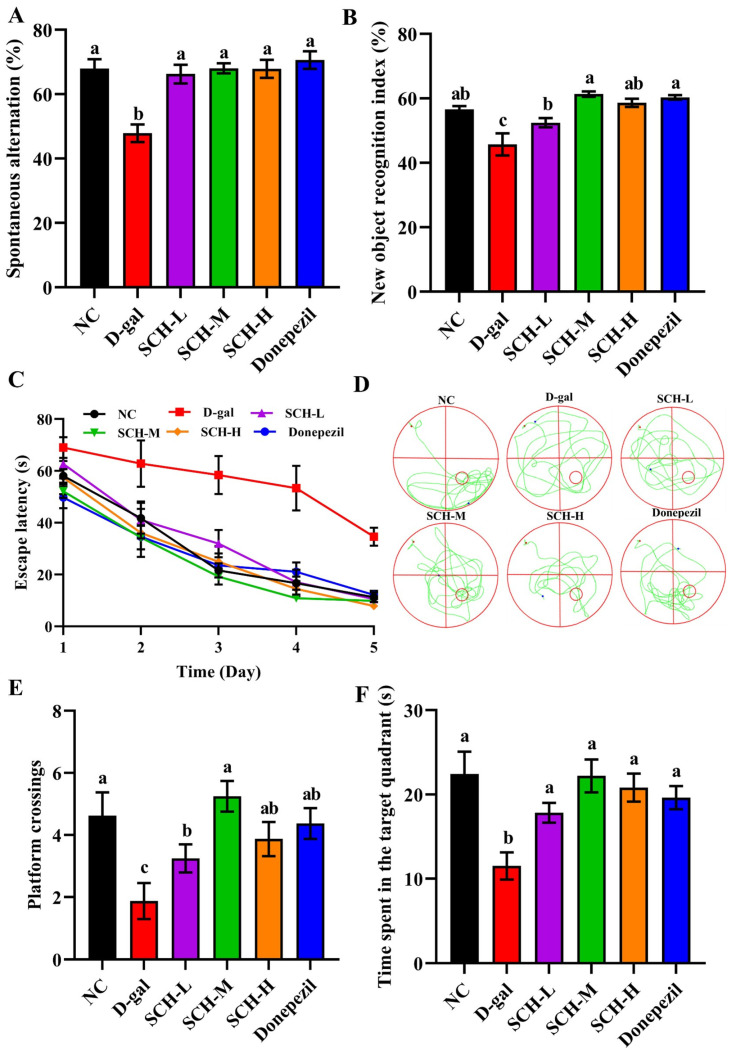
Effects of sea cucumber hydrolysates on cognitive behaviors in aging mice. (**A**) Spontaneous alternation in Y maze test. (**B**) Recognition index in new object recognition test. (**C**) Escape latency during training stage, (**D**) representative trajectory, (**E**) platform crossings number, and (**F**) time on target quadrant in space exploration test of Morris water maze. Letters correspond to significant difference (*p* < 0.05) (*n* = 8–10). SCH-L/M/H: low/medium/high-dose sea cucumber hydrolysates.

No notable differences were observed regarding body weight and food intake among six groups (Supplement Figure S1A,B). In the Y maze test, spontaneous alternation was reduced in the D-gal group than that in the NC group. However, different doses of SCH and donepezil supplementations increased spontaneous alternations (Figure 2A). Similarly, in the novel object recognition test, the D-gal group showed lower recognition index, whereas different doses of SCH and donepezil supplementations elevated the recognition index (Figure 2B). And the effect of donepezil was better than that of the low dose of SCH. During the navigation test, D-gal group exhibited prolonged escape time to arrive at the platform compared to the D-gal group throughout the whole training days (Figure 2C). In the probe trial, the D-gal group exhibited the more disordered trajectory, and spent less time at the target quadrant. However, SCH and donepezil supplementations reversed these trends (Figure 2D–F).

### 3.3. SCH Supplementation Reduced Systemic Inflammation and Enhanced Antioxidant Capacity in Aging Mice

Serum proinflammatory cytokines levels of IL-6, IL-1β, LPS, and TNF-α were enhanced in the D-gal group compared to the NC group. However, supplementations of SCH at various doses, as well as donepezil, reduced levels of TNF-α, LPS, and IL-6. Specifically, SCH-M, SCH-H, and donepezil supplementations reduced the serum IL-1β level. However, the inhibitory effect of donepezil on serum LPS was less than that of medium and high doses of SCH. Concurrently, SCH intervention reversed aging-related antioxidant depletion. Lower serum SOD and anti-inflammatory cytokine such as IL-10 levels were found in the D-gal group. However, SCH and donepezil supplementations increased levels of these cytokines compare to the D-gal group and donepezil showed intermediate efficacy, outperforming low-dose SCH. Notably, SCH-M and SCH-H groups showed similar cytokine levels with the NC group (Table 2).

### 3.4. SCH Supplementation Improved Hippocampal Morphology in Aging Mice

**Figure 3:**
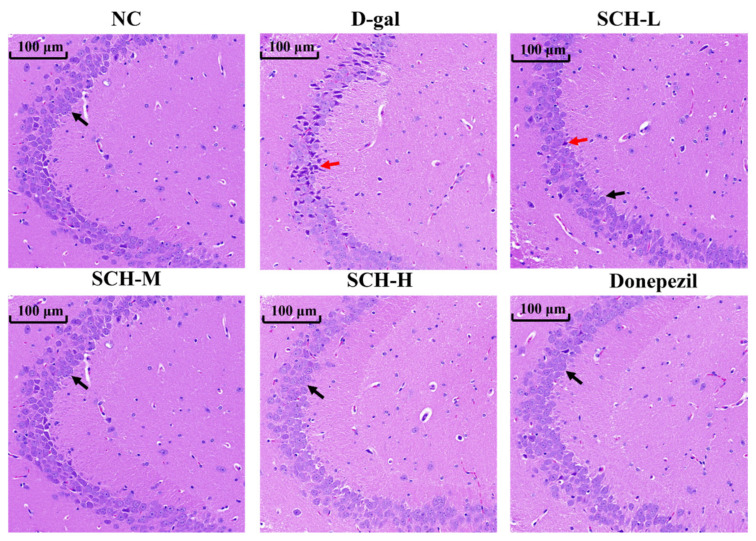
Sea cucumber hydrolysates supplementation improved hippocampal morphology in aging mice. Black arrows represent neurons with normal staining and clear structure; red arrows represent shrinking neurons (*n* = 6). SCH-L/M/H: low/medium/high-dose sea cucumber hydrolysates.

In the NC group, hippocampal neurons exhibited dense populations with well-organized spatial arrangements (black arrows). Neuronal cytoarchitecture remained intact, displaying distinct cellular outlines and uniform nuclear staining intensity. While the D-gal group demonstrated the reduced number of hippocampal neurons, characterized by a loose arrangement, shrunken cells with irregular shapes, and hyperchromatic staining (red arrows). Compared to the D-gal group, SCH-L, SCH-M, SCH-H, and donepezil groups showed the elevated number of hippocampal neurons. Concomitantly, there was a more orderly arrangement and regular morphology, with less hyperchromatic staining (Figure 3).

### 3.5. SCH Supplementation Altered Gut Microbiota Diversity and Composition in Aging Mice

**Figure 4:**
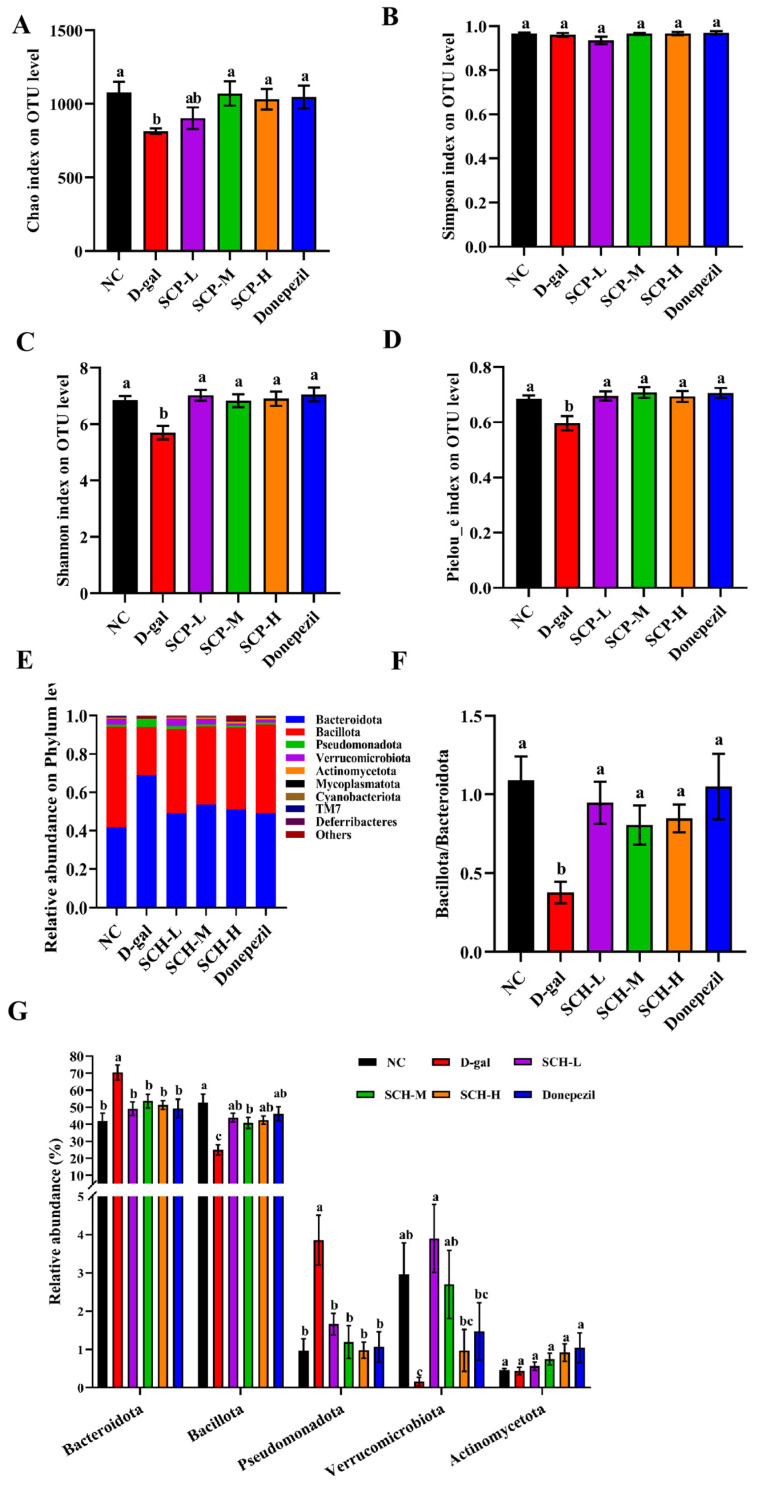

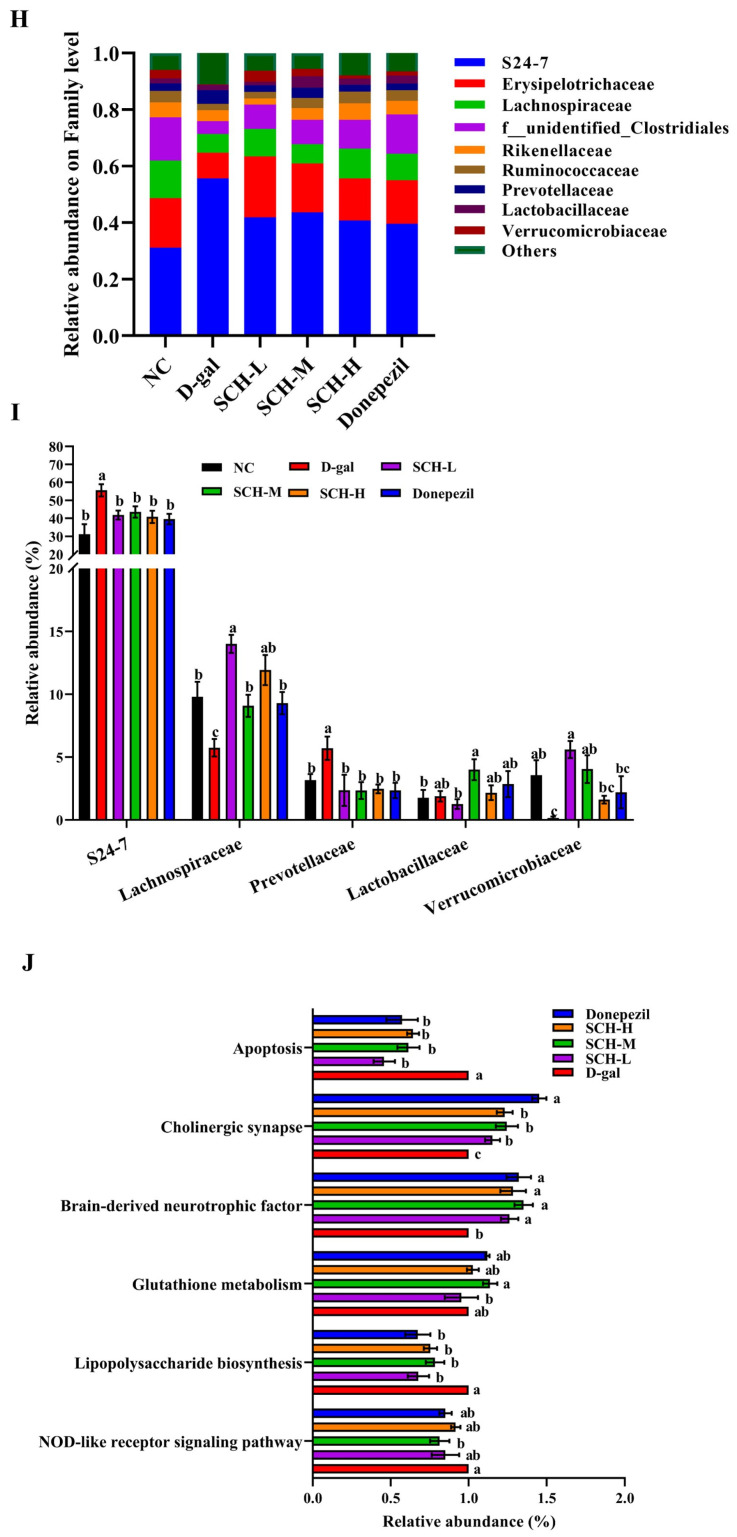
Sea cucumber hydrolysates supplementation altered gut microbiota diversity and composition in aging mice. (**A**) Chao, (**B**) Simpson, (**C**) Shannon, and (**D**) Pielou_e index at OTU level reflected community richness of gut microbiota. (**E**–**G**) Phylum-level, and (**H**,**I**) family-level taxonomic distributions of the microbial communities in feces. (**J**) Comparison of gut microbiota function prediction. KEGG pathway (level 3) was compared by PICRUSt-predicted among all groups. Letters correspond to significant difference (*p* < 0.05) (*n* = 5–6). SCH-L/M/H: low/medium/high-dose sea cucumber hydrolysates.

Community richness was calculated using the Chao index, diversity was measured via Shannon and Simpson indexes, and evenness was measured by the Pielou_e index, as shown in Figure 4A–D. The Chao, Shannon, and Pielou_e indexes were lower in the D-gal group than the NC group. Nevertheless, different doses of SCH and donepezil supplementations increased the Shannon and Pielou_e indices, with the SCH-M, SCH-H, and donepezil groups normalizing the Chao index. However, the Simpson index did not differ among these groups.

At the Phylum-level, compared to the NC group, the D-gal group had lower *Bacillota/Bacteroidota* ratio and levels of *Bacillota* and *Verrucomicrobia*. Nevertheless, it had higher relative levels of *Bacteroidota* and *Pseudomonadota*. SCH and donepezil supplementations increased relative levels of *Bacillota* and the *Bacillota/Bacteroidota* ratio, but reduced relative levels of *Bacteroidota* and *Pseudomonadota*. Nevertheless, the effect of donepezil on *Verrucomicrobiota* was not significantly difference compared to that of D-gal group (Figure 4E–G). At the family-level, the D-gal group showed lower relative levels of *Lachnospiraceae* and *Verrucomicrobiaceae*, while it showed higher relative levels of *Muribaculaceae (S24-7)* and *Prevotellaceae*. SCH and donepezil supplementation reduced relative levels of *S24-7* and *Prevotellaceae*, whereas SCH and donepezil supplementations increased relative levels of *Lachnospiraceae*. Moreover, SCH-L and SCH-M elevated the abundance of *Verrucomicrobiaceae* at the family level. Notably, donepezil showed limited effects on increasing the abundance of *Verrucomicrobiaceae* compared to SCH-L and SCH-M groups (Figure 4H,I).

PICRUSt analysis was further performed to predict gene functional profiles of bacterial communities and characterize the composition of Level 3 KEGG pathways within the bacterial population. The gene abundances in pathways of apoptosis and LPS biosynthesis were decreased in all doses of SCH and donepezil groups. Additionally, the nucleotide-binding oligomerization domain (NOD)-like receptor pathway abundance was decreased in the SCH-M group relative to the D-gal group. Conversely, the gene abundances in pathways of cholinergic synapse and BDNF were increased in different doses of SCH and donepezil groups compared to the D-gal group. There existed no obvious differences in the abundance of glutathione metabolism pathways among all groups. Notably, donepezil demonstrates superior regulatory efficacy at cholinergic synapses compared to SCH, primarily through its ability to enhance cholinergic neurotransmission via AChE inhibition (Figure 4J).

### 3.6. SCH Supplementation Elevated SCFAs Levels in the Feces of Aging Mice

**Figure 5:**
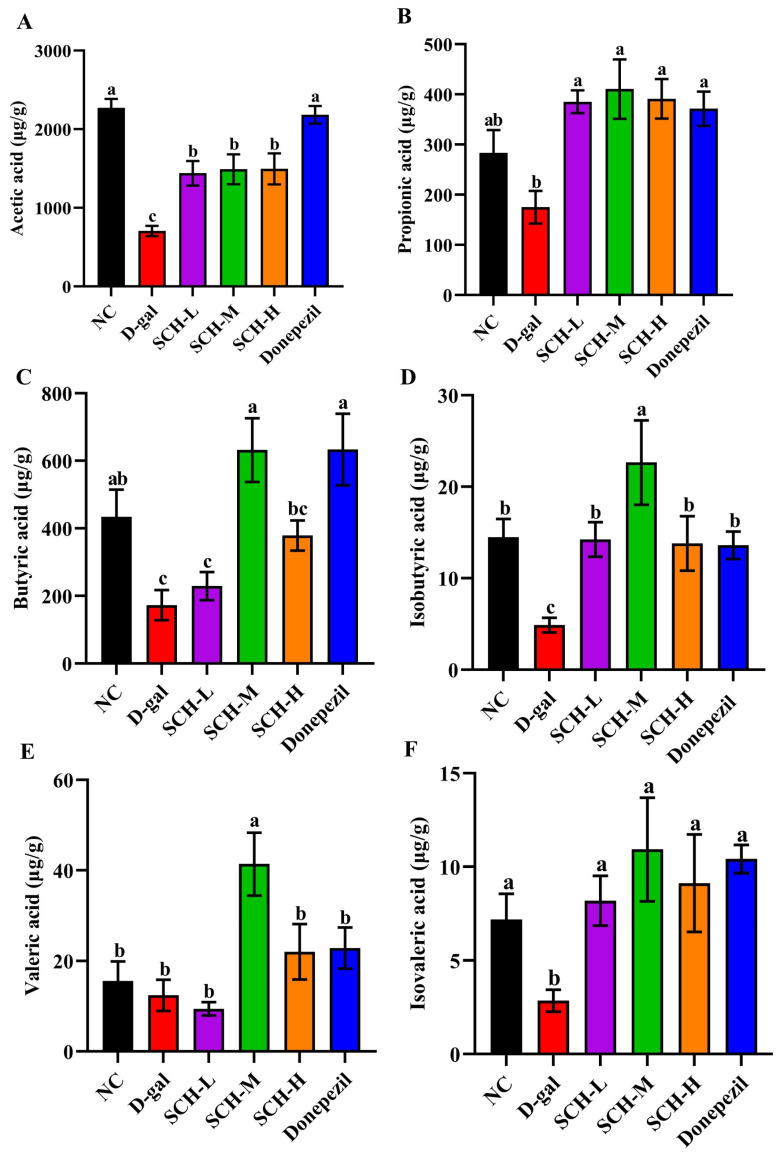
Sea cucumber hydrolysates supplementation elevated fecal SCFAs levels in aging mice. Levels of acetic acid (**A**), propionic acid (**B**), butyric acid (**C**), isobutyric acid (**D**), valeric acid (**E**), and isovaleric acid (**F**) in feces. Letters correspond to significant difference (*p* < 0.05) (*n* = 5–6). SCH-L/M/H: low/medium/high-dose sea cucumber hydrolysates.

The D-gal group exhibited significant reductions in fecal levels of isovaleric acid, acetic acid, and butyric acid. However, SCH-L, SCH-M, SCH-H, and donepezil supplementations increased fecal acetic acid and isovaleric acid levels compared to the D-gal group. Notably, both the medium dose of SCH and donepezil supplementations increased levels of butyric acid level in feces. Furthermore, the medium dose of SCH led to an increased fecal valeric acid level, while varying doses of SCH supplementation resulted in elevated propionic acid levels (Figure 5).

### 3.7. SCH Supplementation Ameliorated Cholinergic Dysfunction in Aging Mice

**Figure 6:**
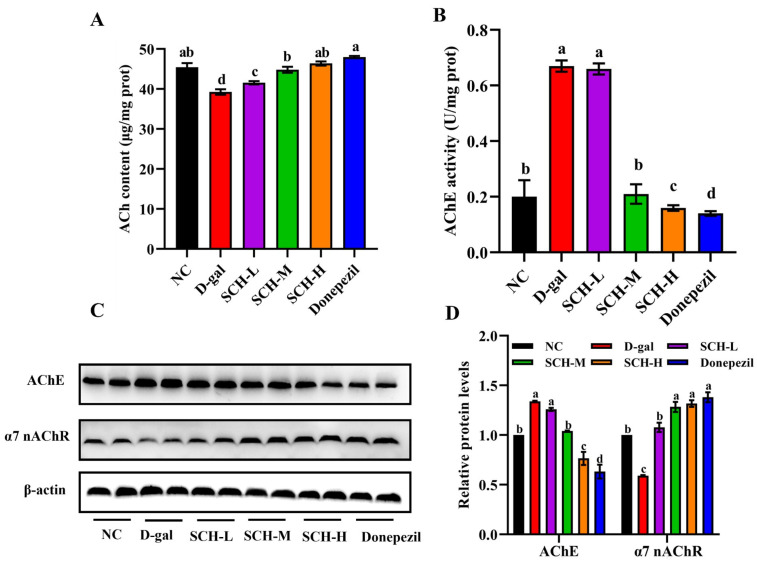
Sea cucumber hydrolysates supplementation ameliorated cholinergic dysfunction in aging mice. (**A**) ACh contents and (**B**) AChE activity in hippocampus. (**C**) Protein expression levels of AChE and α7 nAChR in hippocampus and (**D**) quantification data. Letters correspond to significant difference (*p* < 0.05) (*n* = 6). SCH-L/M/H: low/medium/high-dose sea cucumber hydrolysates; ACh: acetylcholine, α7 nAChR: α7 nicotinic ACh receptor, AChE: acetylcholinesterase.

The hippocampal ACh content was decreased in the D-gal group compared to the NC group. Nevertheless, the D-gal group demonstrated a reduction in hippocampal ACh content compared to NC controls. Notably, both high-dose SCH and donepezil interventions effectively ameliorated this deficit. Concomitant with these findings, the hippocampal AChE activity was elevated in the D-gal group compared to the NC group, which was significantly attenuated by SCH-M, SCH-H, and donepezil supplementations. Meanwhile, the hippocampal protein level of AChE was higher in the D-gal group, while medium and high doses of SCH and donepezil supplementation decreased the hippocampal protein level of AChE. Furthermore, the D-gal group exhibited the decreased hippocampal protein level of α7 nAChR, which was restored by different doses of SCH and donepezil supplementation. In this part of the results, donepezil demonstrated superior regulatory effects on the expression of critical enzymes and proteins within the cholinergic system compared to SCH, primarily due to its selective inhibition of AChE (Figure 6).

### 3.8. SCH Supplementation Activated the BDNF/TrkB Pathway in Aging Mice

**Figure 7:**
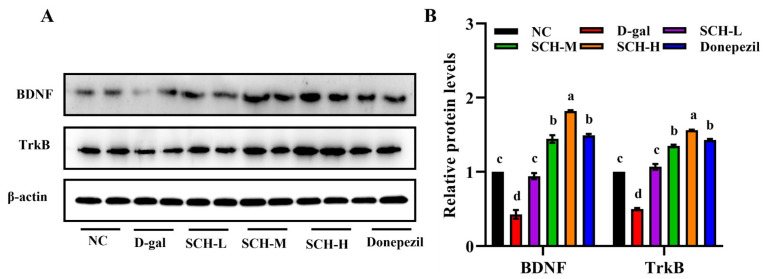
Sea cucumber hydrolysates supplementation activated BDNF/TrkB signaling pathway in aging mice. (**A**) Protein expression levels of BDNF and TrkB in hippocampus and (**B**) quantification data. Letters correspond to significant difference (*p* < 0.05) (*n* = 6). SCH-L/M/H: low/medium/high-dose sea cucumber hydrolysates; TrkB: tropomyosin receptor kinase B; BDNF: brain-derived neurotrophic factor.

The hippocampal protein levels of BDNF and TrkB were decreased in the D-gal group. However, intervention with different doses of SCH dose-dependently elevated hippocampal protein levels of BDNF and TrkB the D-gal group. Similarly, the donepezil group showed elevated protein levels of BDNF and TrkB relative to the D-gal group, while the effect was not superior to the high dose of SCH (Figure 7).

### 3.9. SCH Supplementation Suppressed Neuroinflammation in Aging Mice

**Figure 8:**
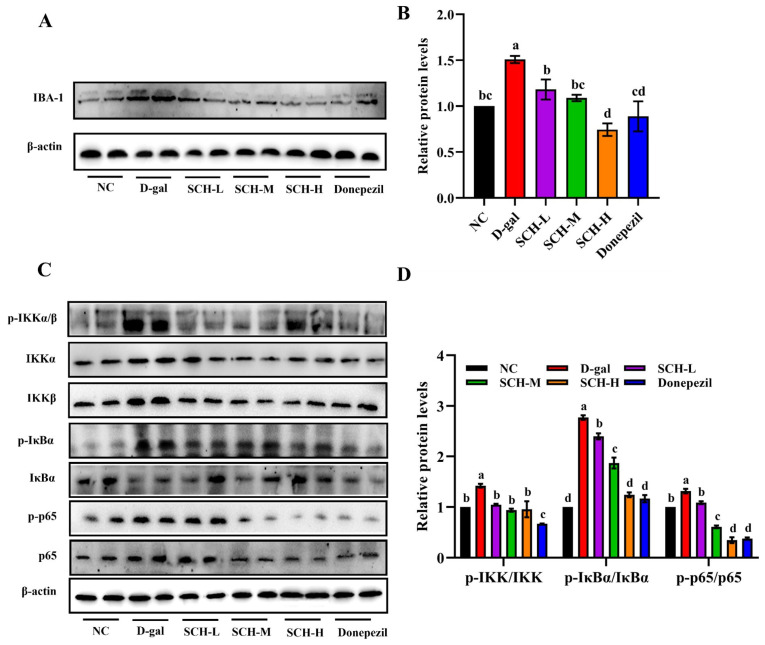
Sea cucumber hydrolysates supplementation suppressed neuroinflammation in D-galactose-induced aging mice. (**A**) Protein expression level of IBA-1 in hippocampus and (**B**) quantification data. (**C**) Protein expression levels of p-IKK/IKK, p-p65/p65, and p-IκBα/IκBα in hippocampus and (**D**) quantification data. Letters correspond to significant difference (*p* < 0.05) (*n* = 6). SCH-L/M/H: low/medium/high-dose sea cucumber hydrolysates; IBA-1: ionized calcium-binding adapter molecule 1; IKK: inhibitor of kappa B kinase; IκBα: inhibitor kappa B alpha.

The D-gal group exhibited the increased hippocampal protein expression of IBA-1 compared to the NC group, indicating enhanced microglial reactivity Nevertheless, SCH and donepezil supplementations decreased protein levels of IBA-1 compared to the D-gal group (Figure 8A,B). The hippocampal protein levels of p-IKK/IKK, p-IκBα/IκBα, and p-p65/p65 were elevated in the D-gal group compared to that in the NC group. Nevertheless, the expression of p-IKK/IKK, p-p65/p65, and p-IκBα/IκBα was reduced after SCH supplementations at different doses, with a similar decrease observed following donepezil supplementation, and the effect was better than that of the low- and medium-dose SCH groups, but comparable to that of the high-dose group (Figure 8C,D).

## 4. Discussion

The D-galactose caused aging model has been widely validated to replicate dementia-associated cognitive deficits, notably including hippocampal damage and memory impairment [27]. Here, behavioral assessments and hippocampal morphology demonstrated that SCH supplementation reversed these aging-related cognitive declines in mice. Emerging evidence highlights the critical role of gut microbiota composition in cognitive health. Notably, the altered *Bacillota/Bacteroidota* ratio linked to cognitive impairment. For instance, walnut-derived peptide ameliorated cognitive impairments while increasing the relative abundance of *Bacillota* in the feces of APP/PS1 mice [28]. However, a fiber-deprived diet increased the phylum *Pseudomonadota*, thereby resulting in hippocampal microglia-mediated synaptic loss and cognitive deficits [29]. Hydrolysates from Atlantic salmon (*Salmo salar*) improves aging-associated neuroinflammation by decreasing *Pseudomonadota* [30]. Additionally, environmental enrichment reversed object recognition impairment via decreasing cytochrome C oxidase activity of the brain in high-fat consumption rats, which coincided with a rise in *Verrucomicrobiota* levels [31], a phenomenon similarly observed in sea cucumber peptide-treated hyperuricemic mice [32]. *Muribaculaceae*, also known as *S24-7*, was correlated with anxiety-like behaviors and increased in hepatic encephalopathy mouse models [33], and responding to cognitive-enhancing interventions like Ginkgolide B [34]. In the previous study, gut microbiota transplant from aging mice affected behavior, and modulated synaptic plasticity in the hippocampus, alongside reductions in *Lachnospiraceae* in the young mice [35]. However, in terms of the Alpha diversity index, the Simpson index, which reflects species dominance, did not differ among these groups. This suggested that SCH may preferentially regulate specific microbial taxa rather than broadly altering community dominance. In our study, aging mice exhibited gut dysbiosis characterized by reduced *Bacillota/Bacteroidota* ratio, decreased *Verrucomicrobiota* and *Lachnospiraceae*, and elevated the level of *Bacteroidota*, *Pseudomonadota*, and *S24-7*. SCH intervention reversed these shifts. Overall, the above results suggested that SCH’s ability to alleviate cognitive decline in aging mice may be related to the regulation of the gut microbiota.

Due to the pivotal role of gut microbiota in the nervous system, this study investigated the predicted functions of microbiota following SCH and donepezil supplementation. NOD-like receptors (NLRs), as critical components of the innate immune system, are responsible for recognizing pathogen-associated molecular patterns. Upon activation, NLRs interact with adaptor proteins to initiate inflammatory signaling pathways such as NF-κB [36,37]. Under physiological conditions, NF-κB p65 remains sequestered in the cytoplasm through binding with its inhibitor IκBα [38]. Upon stimulation, IκBα is degraded by IKK, releasing NF-κB p65, which translocates to the nucleus and activates inflammatory gene transcription and drives cognitive decline [39]. For instance, *Coreopsis tinctoria* essential oil improved cognitive ability via inhibiting NF-κB activation and reducing neuroinflammatory markers including IKKβ, TNF-α, and IL-1β in aging models [40]. Moreover, overexpression of NOD2 could activate NF-κB signaling, counteracting shikonin’s anti-neuroinflammatory efficacy [41]. Notably, there were no obvious differences in the abundance of glutathione metabolism pathways among all groups, implying that SCH’s antioxidative effects in the aging model may be mediated through alternative mechanisms. Consistent with these findings, SCH decreased the level of p-IκBα/IκBα, p-IKK/IKK, and p-p65/p65, thereby blocking the NF-κB signaling and neuroinflammation.

The cholinergic system, encompassing acetylcholine (ACh), cholinergic receptors, acetylcholinesterase (AChE), and BDNF/TrkB signaling, serves as a critical regulator of both cognitive processes and neuroinflammatory modulation [42,43]. Central to this regulatory network, ACh-mediated activation of α7 nAChR suppressed pro-inflammatory cytokines levels and enhanced the IL-10 level, thereby blocking NF-κB translocation and microglial activation [44,45]. Emerging evidence suggests dietary protein hydrolysates can modulate this system, as demonstrated by whey derivatives reducing cerebral AChE activity and inflammation in cognitive impairment models [46], and oyster peptides restoring ACh levels while attenuating glial inflammation in zebrafish [47]. In this study, SCH mitigated cholinergic dysfunction by elevating hippocampal ACh levels and suppressing AChE activity, consistent with prior findings that AChE inhibitors reduced neuroinflammation through modulated cholinergic signaling [48]. Concurrently, SCH enhanced BDNF/TrkB signaling, which synergistically inhibited neuroinflammation by modulating microglial polarization, as evidenced by reduced IBA-1 expression and inhibiting NF-κB signaling [49,50]. *Theragra chalcogramma* and oat protein-derived peptides could alleviate cognitive deficits by enhancing BDNF signaling, accompanied by suppression of neuroinflammation [51,52]. Thus, the cholinergic system homeostasis and BDNF signaling transmission might contribute to suppressing neuroinflammation through inhibition of NF-κB pathway and IBA-1, thereby alleviating cognitive impairment in aging mice after SCH supplementation.

Emerging evidence underscores the critical interface between gut microbiota-derived SCFAs and neuroinflammatory regulation, representing a novel frontier in cognitive research [53]. For instance, mannan oligosaccharide alleviated behavioral disorders, regulated intestinal microecology, and increased fecal SCFAs in 5xFAD mice [54], while Atlantic salmon derived protein hydrolysates attenuated cognitive decline by inhibiting neuroinflammation, which was associated with the increased fecal levels of SCFA in aged rats [30]. In addition, both oral administration and intraperitoneal injection of SCFAs improved learning and cognitive function in mice demonstrated therapeutic potential in murine models, attenuating tau hyperphosphorylation and neuroinflammation while enhancing cognitive performance [55]. This bioavailability is facilitated by SCFAs’ ability to traverse the blood–brain barrier, directly influencing microglial dynamics and NF-κB signaling within the central nervous system [56]. Particularly instructive are findings that dietary butyrate alleviated chronic alcoholic-induced neuronal injury and improved hippocampal pathomorphology through modulating gut microbiota and suppressing microglia-mediated neuroinflammation by the NF-κB signaling pathway [57]. Consistent with these reports, SCH supplementation could reduce serum inflammatory cytokines and IBA-1 and NF-κB pathway-related protein expression in the hippocampus, and these neuroprotective effects were coincided with elevated fecal SCFAs levels observed in the intervention group, suggesting a potential association between modulation of gut microbiota-derived SCFAs and modulation of neuroinflammation after SCH supplementation.

Structure–activity relationship studies reveal critical determinants of neuroactive peptides. Previous researches have demonstrated that walnut-derived peptides with molecular weights <3 kDa exhibited enhanced bioavailability, while those containing Gly sequences showed superior efficacy in ameliorating LPS-induced memory deficits in murine models. Food-derived peptides containing Arg and Asn could bind to the sites of AChE to inhibit its activity, thereby increasing ACh levels to alleviate cognitive decline. Glu, the anion of glutamic acid, is the main excitatory neurotransmitter in the central nervous system, with its neuroprotective effects mediated through specialized astrocytic transport systems that facilitate blood–brain barrier crossing [16,58]. Additionally, hydrophobic amino acids such as Leu, Phe, Val, and Pro and positively charged residues like Lys and Arg have been identified as critical contributors to neuroprotective activity [59,60], while high levels of hydrophobic amino acids such as Gly, Glu, Arg, and Pro have been proven to improve the inflammatory response by regulating the gut microbiota [61,62,63]. Our compositional analysis revealed SCH exhibited significant enrichment of low-molecular-weight peptides and abundant amounts of Gly, Glu, Pro, and Arg. Thus, the molecular weight and composition of SCH could underscore its biological functions in improving cognition.

Our comparative analysis revealed distinct therapeutic profiles between donepezil and SCH interventions. Regarding their differential effects, donepezil, as a selective AChE inhibitor, significantly outperformed SCH in enhancing hippocampal ACh content and suppressing AChE activity, which aligns with its pharmacological mechanism of action. However, medium/high-dose of SCH achieved comparable anti-inflammatory efficacy to donepezil, significantly reducing both systemic cytokines and hippocampal neuroinflammatory markers. Notably, compared with donepezil, high dose of SCH had a more significant activation effect on the BDNF/TrkB pathway, which may be attributed to the specific regulation of anti-inflammatory and neurotrophic signals by low-molecular-weight peptides in SCH. And SCH demonstrated a broader regulatory effect on gut microbiota. These differential effects position SCH as a multimodal intervention combining in regulating the gut microbiota, activating the BDNF pathway and suppressing neuroinflammation through the synergistic effect of multiple peptides. When comparing SCH doses, the medium dose of SCH showed superior efficacy in regulating specific behavior and SCFAs and inhibiting neuroinflammatory pathways, while also modulating the BDNF pathway and the cholinergic system. Although a high dose of SCH was superior in activating the BDNF pathway, a medium dose equivalently improved behavioral parameters, hippocampal morphology, gut microbiota composition, and SCFA production. Considering both effect and cost, the medium dose is the most suitable one.

While our findings highlighted the potential of SCH in mitigating cognitive deficits in a D-galactose-induced aging model, three key limitations warrant consideration. First of all, while our study revealed correlations between SCH supplementation, gut microbiota, microbiota-derived SCFAs, neuroprotective effect, behavior, and neuroinflammatory markers, definitive causal relationships require validation through targeted microbial interventions. The hypotheses for future research are as follows: (1) SCH-enhanced SCFAs may traverse the blood–brain barrier to inhibit NF-κB-dependent microglial activation; (2) Microbiota remodeling may concurrently potentiate cholinergic signaling and BDNF/TrkB neurotrophic pathways, creating synergistic anti-inflammatory effects. To test this model, three-pronged approaches are recommended including fecal microbiota transplantation, antibiotic-induced microbiota depletion, and metabolomic profiling of SCH-induced microbial metabolites with subsequent pathway validation in gnotobiotic models. Then, the study employed a murine model of drug-induced aging, which may not fully recapitulate the complexity of human aging or specific neurodegenerative diseases such as Alzheimer’s disease. Thus, other models of cognitive decline and clinical trials are required in the future. In addition, this study focused only on the effect of SCH on male aging mice and the results might be limited to males. Further research is needed to reveal the sex-based differences in the positive effects of SCH on aging-related cognitive issues.

## 5. Conclusions

In total, this research demonstrated that supplementation with SCH alleviated behavioral deficits and hippocampal pathology in aging mice. Additionally, SCH modulated gut microbiota, along with increased levels of fecal SCFAs. Functional prediction revealed that alterations in gut microbiota were correlated with signal transduction in the hippocampus. Third, regulation involving cholinergic system restoration and BDNF/TrkB neurotrophic activation was mechanistically linked to neuroinflammatory suppression through NF-κB pathway inhibition. These findings suggest that SCH could be a natural strategy to counter cognitive deficits, which might be related to regulating gut microbiota. These results highlight that SCH can be considered a powerful dietary supplement that may be helpful for alleviating aging-related cognitive deficits.

## Figures and Tables

**Table 1:** Amino acid composition of SCH. (*n* = 3). SCH: sea cucumber hydrolysates.

Amino Acid	g/100 g Hydrolysates
Asp	5.30 ± 0.11
Glu	8.85 ± 0.08
Ser	2.20 ± 0.05
His	0.52 ± 0.07
Gly	11.17 ± 0.09
Thr	2.34 ± 0.06
Arg	5.91 ± 0.06
Ala	5.76 ± 0.12
Tyr	0.90 ± 0.01
Cys-s	0.03 ± 0.00
Val	2.45 ± 0.10
Met	1.10 ± 0.04
Phe	1.84 ± 0.03
Ile	1.63 ± 0.14
Leu	2.38 ± 0.10
Lys	1.98 ± 0.06
Pro	6.78 ± 1.14

**Table 2:** Sea cucumber hydrolysates supplementation reduced systemic inflammation and enhanced antioxidant capacity in aging mice.

Parameters	NC	D-gal	SCH-L	SCH-M	SCH-H	Donepezil
Serum SOD (U/mL)	86.59 ± 1.31 ^b^	74.17 ± 1.18 ^d^	80.05 ± 1.47 ^c^	88.76 ± 1.61 ^ab^	93.42 ± 4.01 ^a^	92.04 ± 2.58 ^ab^
Serum LPS (pg/mL)	130.00 ± 9.2 ^c^	268.38 ± 13.56 ^a^	203.23 ± 15.88 ^b^	130.00 ± 11.44 ^c^	150.20 ± 8.98 ^c^	208.79 ± 5.74 ^b^
Serum IL-6 (pg/mL)	23.88 ± 0.65 ^c^	30.83 ± 0.91 ^a^	27.81 ± 0.91 ^b^	27.26 ± 1.44 ^c^	24.42 ± 1.23 ^c^	26.77 ± 0.27 ^bc^
Serum IL-1β (pg/mL)	79.92 ± 2.21 ^b^	97.56 ± 2.10 ^a^	91.58 ± 2.21 ^a^	76.17 ± 1.88 ^b^	80.06 ± 3.01 ^b^	81.86 ± 3.01 ^b^
Serum TNF-α (pg/mL)	22.08 ± 1.62 ^bc^	31.28 ± 1.33 ^a^	26.88 ± 0.81 ^b^	21.75 ± 1.00 ^c^	22.75 ± 1.00 ^bc^	25.68 ± 0.87 ^bc^
Serum IL-10 (pg/mL)	28.24 ± 2.17 ^a^	14.02 ± 1.74 ^c^	20.86 ± 0.89 ^b^	31.19 ± 2.02 ^a^	29.41 ± 2.94 ^a^	30.24 ± 3.44 ^a^

Letters correspond to significant difference (*p* < 0.05) (*n* = 6). SCH-L/M/H: low/medium/high-dose sea cucumber hydrolysates; SOD: superoxide dismutase; IL-10: interleukin-10; TNF-α: tumor necrosis factor-α; IL-6: interleukin-6; LPS: lipopolysaccharide; IL-1β: interleukin-1β.

## Data Availability

The original contributions presented in this study are included in the article/Supplementary Materials. Further inquiries can be directed to the corresponding author.

## Supplementary Materials

The following supporting information can be downloaded at: https://www.mdpi.com/article/10.3390/foods14111938/s1, Figure S1: Effects of SCH on body weight (A) and food intake (B) in aging mice.
