# Supplementary material for: Sea Cucumber Hydrolysates Alleviate Cognitive Deficits in D-Galactose-Induced C57BL/6J Aging Mice Associated with Modulation of Gut Microbiota
Source: Foods. 2025 May 29;14(11):1938. doi: 10.3390/foods14111938 (PMC12154004; doi:10.3390/foods14111938)

Supplement Figure

Supplement Figure S1. Effects of SCH on body weight (A) and food intake (B) in D-galactose-induced aging mice. The letters correspond to significant difference ($P < 0.05$) (n=6). SCH-L: low dose of sea cucumber hydrolysates; SCH-M: medium dose of sea cucumber hydrolysates; SCH-H: high dose of sea cucumber hydrolysates.

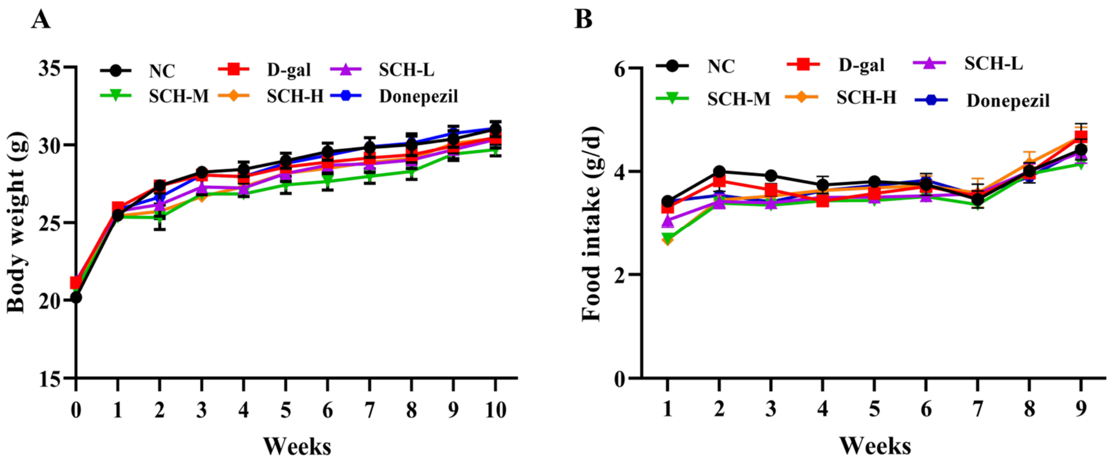

Supplement: Supplementary file 1 [file foods-14-01938-s001.zip › foods-3631133-supplementary.pdf]
